# Supplementary material for: Ageing-associated changes in transcriptional elongation influence longevity
Source: Nature. 2023 Apr 12;616(7958):814–21. doi: 10.1038/s41586-023-05922-y (PMC10132977; doi:10.1038/s41586-023-05922-y)
Supplement: Supplementary file 1 — This file contains Supplementary Fig. 1 and Supplementary Tables 1–3. Supplementary Fig. 1 contains the raw scans of two western blotted membranes from Extended Data Fig. 10a. Each membrane was cut into two pieces (low and high MW) and blotted with different antibodies. The positions of molecular weight (MW) markers are shown alongside each membrane. The parts crossed out correspond to areas of the blot not used in the corresponding figure. Supplementary Table 1 includes descriptions of the RNA-seq datasets used. Supplementary Table 2 contains Pearson correlations between the change in elongation rate and characteristics of the introns in which the elongation rate was measured in the respective RNA-Seq datasets. Supplementary Table 3 includes the sample sizes of statistical comparisons in the main figures. [file 41586_2023_5922_MOESM1_ESM.pdf]

nature portfolio

https://doi.org/10.1038/s41586-023-05922-y

Supplementary information

Ageing-associated changes in transcriptional elongation influence longevity

In the format provided by the authors and unedited

Supplementary Figure 1

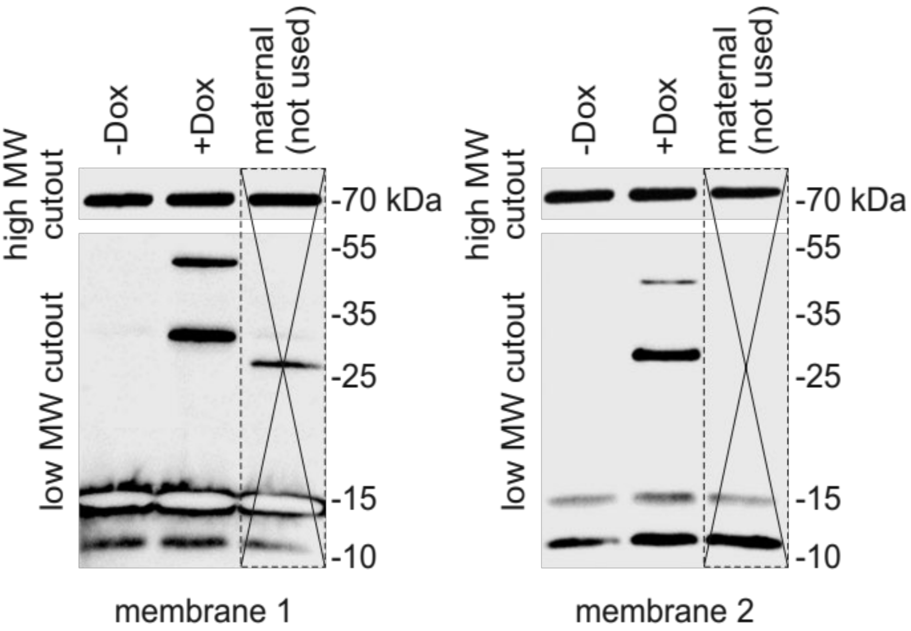

Supplementary Table 1: Description of the RNA-seq datasets used in the study.

**Species**	**Tissue**	**Enrichment protocol**	**Sequencing parameters** Paired-/single-end, read length, millions of reads (M)	**Time points[1] and conditions**
C. elegans	Whole body	TruSeq Stranded Total RNA Library	Paired-end, 75 *bp*, 25 M	Day 1 (*WT*), day 7 (*WT*), day 14 (*WT*), day 21 (*WT*)
	Whole body	TruSeq Stranded Total RNA Library	Paired-end, 75 *bp*, 25 M	Day 14 (*WT*, *daf-2(e1370), ama-1(m322)*)
	Whole Body	TruSeq Stranded Total RNA library	Paired-end, 75 bp, 25 M	Day 1 (WT, ama-1(m322))
D. melanogaster	Head	TruSeq Stranded Total RNA Library	Single-end, 100 *bp*, 37.5 M	Day 30 (*WT*, *dilp2,3-5*), Day 50 (*WT*, *dilp2,3-5*)
	Head	TruSeq Stranded Total RNA Library	Paired-end, 75 *bp*, 30 M	Day 10 (*WT*, $RpII215^{C4}$), day 50 (*WT*, $RpII215^{C4}$)
M. musculus	Kidney	TruSeq Stranded Total RNA Library	Paired-end, 75 *bp*, 70 M	Month 3 (*WT*), month 24 (*WT*)
	Kidney	TruSeq Stranded Total RNA Library	Paired-end, 75 *bp*, 30 M	Month 3 (*WT*, *DR*) (4 replicates)
	Liver	TruSeq Stranded Total RNA Library	Paired-end, 75 *bp*, 37.5 M	Month 5 (*WT*, *DR*)
	Liver	TruSeq Stranded Total RNA Library	Paired-end, 75 *bp*, 37.5 M	Month 16 (*WT*, *DR*)

[1] Triplicate except where mentioned otherwise.

**Species**	**Tissue**	**Enrichment protocol**	**Sequencing parameters** Paired-/single-end, read length, millions of reads (M)	**Time points[1] and conditions**
	Liver	TruSeq Stranded Total RNA Library	Paired-end, 75 *bp*, 37.5 M	Month 27 (*WT*, *DR*)
	Blood	TruSeq Stranded Total RNA Library	Paired-end, 75 *bp*, 70 M	Month 5 *(WT),* month 27 *(WT)*
	Hypothalamu s	TruSeq Stranded Total RNA Library	Single-end, 100 *bp*, 30 M	Month 26 (*WT*, *IRS1-/-*)
R. norvegicus (*17*)	Liver	TruSeq Stranded Total RNA Library	Single-end, 50 *bp*, 60 M	Month 6 (*WT*), month 24 (*WT*) (2 replicates)
	Brain[2]	TruSeq Stranded Total RNA Library	Single-end, 50 *bp*, 20 M	Month 6 (*WT*), month 24 (*WT*) (6 replicates)
H. sapiens	Fetal lungs (IMR90)	Nascent RNA	Paired-end, 75 *bp*, 25 M	Early passage, late passage (2 replicates)
	Fetal lungs (IMR90)	TruSeq Stranded Total RNA Library	Paired-end, 75 *bp*, 50 M	Early passage, late passage (2 replicates)
	Umbilical vein endothelial (HUVECs)	Nascent RNA	Paired-end, 75 *bp*, 50 M	Early passage, late passage (2 replicates)
	Umbilical vein endothelial (HUVECs)	TruSeq Stranded Total RNA Library	Paired-end, 75 *bp*, 100 M	Early passage, late passage

[2] Not included in the analysis due to low coverage (bellow 1X genome coverage or 29 M sequenced reads for *R. norvegicus* ; genome coverage calculated using Lander-Waterman formula)

**Species**	**Tissue**	**Enrichment protocol**	**Sequencing parameters** Paired-/single-end, read length, millions of reads (M)	**Time points[1] and conditions**
	Fibroblast skin	TruSeq Stranded Total RNA Library	Paired-end, 100 *bp*, 100 M	Progeria patient (HSS) (2 replicates)
			Paired-end, 75 *bp*, 100 M	Healthy donor, sex/age matched with progeria patient (2 replicates)
	Blood	TruSeq Stranded Total RNA Library	Paired-end, 75 *bp*, 70 M	Healthy donor, 6 females, 6 males, age range: 21-70

Supplementary Table 2: Pearson correlations between the change in elongation rate and characteristics of the introns in which the elongation rate was measured in the respective RNA-Seq datasets

**Species**	**Comparison**	**Distance from promoter**	**Intron length**	**Gene expression log2FC**	**Circular RNA index**
C. elegans	21 d vs 1 d	0.014	0.071	-0.266	-0.085
C. elegans	14 d ama-1 vs 14 d wt	0.008	-0.110	-0.247	0.160
C. elegans	14 d daf2 vs 14 d wt	0.020	0.007	-0.278	0.130
D. melanogaster	50 d vs 10 d	-0.036	0.019	-0.023	0.019
D. melanogaster	50 d RpII215 vs 50 d	0.002	-0.091	-0.161	-0.043
D. melanogaster	50 d dilp 2,3-5 vs 50 d	-0.133	-0.170	0.041	-0.092
M. musculus	24 mo vs 3.5 mo	0.011	0.043	-0.230	-0.045
H. sapiens	Senescent vs proliferating (IMR90)	-0.021	0.046	-0.274	0.184
H. sapiens	Senescent vs proliferating (HUVEC)	0.014	0.036	-0.289	0.020

Supplementary Table 3: Sample size of statistical comparisons in the main figures.

Species	Comparison	Fig. 1c/ Fig. 2a (All introns)	Fig. 1c/ Fig. 2a (Consistent introns)	Fig. 3a	Fig. 3b	Fig. 3c	Fig. 3d (Genes)
C. elegans	14 d vs 1 d	576	115	2701	335	7096	5752
C. elegans	21 d vs 1 d	520	129	2451	335	7096	5752
C. elegans	14 d ama-1 vs 14 d wt	594	176	2404	3702	5849	6238
C. elegans	1 d ama-1 vs 1 d wt	557	99	-	3702	-	6238
C. elegans	14 d wt vs 1 d wt	436	107	-	-	-	6238
C. elegans	14 d daf2 vs 14 d wt	518	69	2257	3702	-	6238
D. melanogaster	50 d vs 30 d	531	78	-	120	-	-
D. melanogaster	50 d RpII215 vs 50 d	1233	257	1638	2653	4952	2727
D. melanogaster	10 d RpII215 vs 10 d	1297	374	-	2653	4952	2727
D. melanogaster	50 d RpII215 vs 10 d RpII215	1316	299	-	2653	-	
D. melanogaster	50 d wt vs 10 d wt	1200	209	1676	2653	4952	
D. melanogaster	10 d dilp 2,3-5 vs 10 d	546	77	-	120	-	-
D. melanogaster	50 d dilp 2,3-5 vs 50 d	527	153	-	120	-	-
M. musculus	Blood (Old vs. young)	1559	152	4546	393	41004	1950
M. musculus	DR (5 weeks)	3280	625	-	-	-	3441

**Species**	**Comparison**	**Fig. 1c/ Fig. 2a (All introns)**	**Fig. 1c/ Fig. 2a (Consistent introns)**	**Fig. 3a**	**Fig. 3b**	**Fig. 3c**	**Fig. 3d (Genes)**
M. musculus	DR (16 weeks)	3636	609	-	-	-	3441
M. musculus	DR (26 weeks)	3586	629	-	-	-	3441
M. musculus	Caloric restriction, kidney	3270	148	-	1952	-	2171
M. musculus	24 mo vs 3.5 mo	4455	597	1457	2486	13297	5674
M. musculus	26 mo IRS vs 26 mo	5555	390	711	2200	-	-
R. norvegicus	24 mo vs. 6 mo	6384	2095	376	-	-	-
H. sapiens	Blood (Old vs. young)	6746	57	6120	2166	28683	4969
H. sapiens	Senescent vs Proliferating (IMR90)	6969	2988	773	4932	1121	3403
H. sapiens	Senescent vs Proliferating (HUVEC)	6497	741	8486	6283	26701	8620
