# Supplementary material for: Ageing-associated changes in transcriptional elongation influence longevity
Source: Nature. 2023 Apr 12;616(7958):814–21. doi: 10.1038/s41586-023-05922-y (PMC10132977; doi:10.1038/s41586-023-05922-y)
Supplement: Supplementary file 2 — Reporting Summary [file 41586_2023_5922_MOESM2_ESM.pdf]

nature research

Corresponding author(s): Andreas Beyer

Last updated by author(s): Feb 25, 2023

Reporting Summary

Nature Research wishes to improve the reproducibility of the work that we publish. This form provides structure for consistency and transparency in reporting. For further information on Nature Research policies, see Authors & Referees and the Editorial Policy Checklist.

Statistics

For all statistical analyses, confirm that the following items are present in the figure legend, table legend, main text, or Methods section.

n/a	Confirmed	
☐	☒	The exact sample size (n) for each experimental group/condition, given as a discrete number and unit of measurement
☐	☒	A statement on whether measurements were taken from distinct samples or whether the same sample was measured repeatedly
☐	☒	The statistical test(s) used AND whether they are one- or two-sided<br>*Only common tests should be described solely by name; describe more complex techniques in the Methods section.*
☒	☐	A description of all covariates tested
☐	☒	A description of any assumptions or corrections, such as tests of normality and adjustment for multiple comparisons
☐	☒	A full description of the statistical parameters including central tendency (e.g. means) or other basic estimates (e.g. regression coefficient) AND variation (e.g. standard deviation) or associated estimates of uncertainty (e.g. confidence intervals)
☐	☒	For null hypothesis testing, the test statistic (e.g. F, t, r) with confidence intervals, effect sizes, degrees of freedom and P value noted<br>*Give P values as exact values whenever suitable.*
☒	☐	For Bayesian analysis, information on the choice of priors and Markov chain Monte Carlo settings
☒	☐	For hierarchical and complex designs, identification of the appropriate level for tests and full reporting of outcomes
☐	☒	Estimates of effect sizes (e.g. Cohen's d, Pearson's r), indicating how they were calculated

Our web collection on statistics for biologists contains articles on many of the points above.

Software and code

Policy information about availability of computer code

Data collection	Code details and version are provided in the Methods section. Only open source code was used and references are provided in the Methods section.
Data analysis	Details about the data analysis are provided in the Methods section. Only open source code was used and references are provided in the the Methods section.<br>Newly developed code can be downloaded from here:<br>https://github.com/beyergroup/ElongationRate

For manuscripts utilizing custom algorithms or software that are central to the research but not yet described in published literature, software must be made available to editors/reviewers. We strongly encourage code deposition in a community repository (e.g. GitHub). See the Nature Research guidelines for submitting code & software for further information.

Data

Policy information about availability of data

All manuscripts must include a data availability statement. This statement should provide the following information, where applicable:

- Accession codes, unique identifiers, or web links for publicly available datasets
- A list of figures that have associated raw data
- A description of any restrictions on data availability

Data were uploaded to GEO. Reference is provided in the manuscript.

Field-specific reporting

Please select the one below that is the best fit for your research. If you are not sure, read the appropriate sections before making your selection.

☒ Life sciences ☐ Behavioural & social sciences ☐ Ecological, evolutionary & environmental sciences

For a reference copy of the document with all sections, see nature.com/documents/nr-reporting-summary-flat.pdf

Life sciences study design

All studies must disclose on these points even when the disclosure is negative.

Sample size	All measurements were done in triplicates, unless stated otherwise (see Extended Data Table 2). Results were summarized across large numbers of genes/introns (see figures for details). Thus, even duplicate measurements were sufficient to gain sufficient statistical power. (P-values are provided in the manuscript.)
Data exclusions	No data was excluded. All data was uploaded to GEO.
Replication	All measurements were done in biological replicates (at least twice, mostly three times). The main conclusions were highly consistent across species and tissues, which indicates high reproducibility. The findings regarding splicing changes were reproducible across most, but not all species. We attribute this to the large variation in splicing complexity across species. (The number of alternative isoforms in mammals is vastly larger than in fly and worm.)
Randomization	There was no grouping, i.e. no randomization necessary. In case of the human blood samples we had the same number of male and female subjects included.
Blinding	Since no grouping was done, there was no need for blinding.

Reporting for specific materials, systems and methods

We require information from authors about some types of materials, experimental systems and methods used in many studies. Here, indicate whether each material, system or method listed is relevant to your study. If you are not sure if a list item applies to your research, read the appropriate section before selecting a response.

Materials & experimental systems

n/a	Involved in the study	
☒	☐	Antibodies
☐	☒	Eukaryotic cell lines
☒	☐	Palaeontology
☐	☒	Animals and other organisms
☐	☒	Human research participants
☒	☐	Clinical data

Methods

n/a	Involved in the study	
☒	☐	ChIP-seq
☒	☐	Flow cytometry
☒	☐	MRI-based neuroimaging

Eukaryotic cell lines

Policy information about cell lines

Cell line source(s)	Human umbilical vein endothelial cells (HUVECs) from individual healthy donors were purchased from Lonza Inc.; human primary lung fibroblasts (IMR90) from two different isolates were obtained via the Coriell repository.
Authentication	These lines were authenticated by their commercial provider.
Mycoplasma contamination	All these lines were biannually checked for mycoplasma contamination and tested negative.
Commonly misidentified lines (See ICLAC register)	None identiifed.

Animals and other organisms

Policy information about studies involving animals; ARRIVE guidelines recommended for reporting animal research

Laboratory animals	Mus musculus. Female F1 hybrid mice (C3B6F1) were generated in-house by crossing C3H/HeOuJ females with C57BL/6 NCrl males (strain codes 626 and 027, respectively, Charles River Laboratories).<br><br>Drosophila melanogaster: v[1], RpII215[4] (RRID:BDSC_3663) mutant flies were obtained from the Bloomington Drosophila Stock

Center (NIH P40OD018537). The RpII215[4] allele was backcrossed for 6 generations into the outbred white Dah wild type background (Grönke et al., 2010) generating the w1118, RpII215[4] stock, which was used for experiments. wDah, dilp2-3,5 flies (RRID:BDSC_30889) were previously generated in the lab and backcrossed for 6 generations into the outbred white Dah wild type background (Grönke et al., 2010). Female flies were used for all experiments.

C. elegans strains used: AA4274 ama-1(m322), CB1370 daf-2(e1370), N2 wild type

Wild animals	n/a
Field-collected samples	n/a
Ethics oversight	The DR study was performed in accordance with the recommendations and guidelines of the Federation of the European Laboratory Animal Science Association (FELASA), with all protocols approved by the Landesamt für Natur, Umwelt und Verbraucherschutz, Nordrhein-Westfalen, Germany (reference numbers: 8.87-50.10.37.09.176 and 84-02.04.2015.A437).

Note that full information on the approval of the study protocol must also be provided in the manuscript.

Human research participants

Policy information about studies involving human research participants

Population characteristics	Healthy male and female subjects between 21 and 70 years of age.
Recruitment	Participants were searched using bulletins in which healthy individuals interested in taking part in a study examining aging-related changes were asked to contact the Dept. 2 of Internal Medicine (UoC) by telephone. A trained employee ruled out relevant pre-existing diseases using a structured questionnaire. The test persons were then invited for an appointment at the University Hospital Cologne to obtain the blood samples used for sequencing in the study at hand.
Ethics oversight	Institutional Review Board, Medical Faculty, University of Cologne

Note that full information on the approval of the study protocol must also be provided in the manuscript.
